# On optimal multiple changepoint algorithms for large data

**DOI:** 10.1007/s11222-016-9636-3

**Published:** 2016-02-15

**Authors:** Robert Maidstone, Toby Hocking, Guillem Rigaill, Paul Fearnhead

**Affiliations:** 1 STOR-i Centre for Doctoral Training, Lancaster University, Lancaster, UK; 2 McGill University and Genome Quebec Innovation Center, Quebec, Canada; 3 Institute of Plant Sciences Paris-Saclay, UMR 9213/UMR1403, CNRS, INRA, Université Paris-Sud, Université d’Evry, Université Paris-Diderot, Sorbonne Paris-Cité, Paris, France; 4 Department of Mathematics and Statistics, Lancaster University, Lancaster, UK

**Keywords:** Breakpoints, Dynamic Programming, FPOP, SNIP, Optimal Partitioning, pDPA, PELT, Segment Neighbourhood

## Abstract

Many common approaches to detecting changepoints, for example based on statistical criteria such as penalised likelihood or minimum description length, can be formulated in terms of minimising a cost over segmentations. We focus on a class of dynamic programming algorithms that can solve the resulting minimisation problem exactly, and thus find the optimal segmentation under the given statistical criteria. The standard implementation of these dynamic programming methods have a computational cost that scales at least quadratically in the length of the time-series. Recently pruning ideas have been suggested that can speed up the dynamic programming algorithms, whilst still being guaranteed to be optimal, in that they find the true minimum of the cost function. Here we extend these pruning methods, and introduce two new algorithms for segmenting data: FPOP and SNIP. Empirical results show that FPOP is substantially faster than existing dynamic programming methods, and unlike the existing methods its computational efficiency is robust to the number of changepoints in the data. We evaluate the method for detecting copy number variations and observe that FPOP has a computational cost that is even competitive with that of binary segmentation, but can give much more accurate segmentations.

**Electronic supplementary material:**

The online version of this article (doi:10.1007/s11222-016-9636-3) contains supplementary material, which is available to authorized users.

## Introduction

Often time-series data experiences multiple abrupt changes in structure which need to be taken into account if the data is to be modelled effectively. These changes, known as changepoints, or breakpoints, cause the data to be split into segments which can then be modelled separately. Detecting changepoints, both accurately and efficiently, is required in a number of applications including bioinformatics (Picard et al. 2011), financial data (Fryzlewicz 2012), climate data (Killick et al. 2012; Reeves et al. 2007), EEG data (Lavielle 2005), Oceanography (Killick et al. 2010) and the analysis of speech signals (Davis et al. 2006).

As increasingly large data-sets are obtained in modern applications, there is a need for statistical methods for detecting changepoints that are not only accurate but also are computationally efficient. A motivating application area where computational efficiency is important is in detecting copy number variation (Olshen et al. 2004; Zhang et al. 2010). For example, in Sect. 7 we look at detecting changes in DNA copy number in tumour microarray data. Accurate detection of regions in which this copy number is amplified or reduced from a baseline level is crucial as these regions can relate to tumorous cells and their detection is important for classifying tumour progression and type. The data analysis in Sect. 7 involves detecting changepoints in thousands of time-series, many of which have hundreds of thousands of data points. Other applications of detecting copy number variation can involve analysing data sets which are orders of magnitude larger still.

There are a wide-range of approaches to detecting changepoints, see for example Frick et al. (2014) and Aue and Horvth (2013) and the references therein. We focus on one important class of approaches (e.g. Braun et al. 2000; Davis et al. 2006; Zhang and Siegmund 2007) that can be formulated in terms of defining a cost function for a segmentation. They then either minimise a penalised version of this cost (e.g. Yao 1988; Lee 1995), which we call the penalised minimisation problem; or minimise the cost under a constraint on the number of changepoints (e.g. Yao and Au 1989; Braun and Müller 1998), which we call the constrained minimisation problem. If the cost function depends on the data through a sum of segment-specific costs then the minimisation can be done exactly using dynamic programming (Auger and Lawrence 1989; Jackson et al. 2005). However these dynamic programming methods have a cost that increases at least quadratically with the amount of data, and is prohibitive for large-data applications.

Alternatively, much faster algorithms exist that provide approximate solutions to the minimisation problem. The most widely used of these approximate techniques is Binary Segmentation (Scott and Knott 1974). This takes a recursive approach, adding changepoints one at a time. With a new changepoint added in the position that would lead to the largest reduction in cost given the location of previous changepoints. Due to its simplicity, Binary Segmentation is computationally efficient, being roughly linear in the amount of data, however it only provides an approximate solution and can lead to poor estimation of the number and position of changepoints (Killick et al. 2012). Variations of Binary Segmentation, such as Circular Binary Segmentation (Olshen et al. 2004) and Wild Binary Segmentation (Fryzlewicz 2012), can offer more accurate solutions for slight decreases in the computational efficiency.

An alternative approach is to look at ways of speeding up the dynamic programming algorithms. Recent work has shown this is possible via pruning of the solution space. Killick et al. (2012) present a technique for doing this which we shall refer to as *inequality based pruning*. This forms the basis of their method PELT which can be used to solve the penalised minimisation problem. Rigaill (2010) develop a different pruning technique, *functional pruning*, and this is used in their pDPA method which can be used to solve the constrained minimisation problem. Both PELT and pDPA are optimal algorithms, in the sense that they find the true optimum of the minimisation problem they are trying to solve. However the pruning approaches they take are very different, and work well in different scenarios. PELT is most efficient in applications where the number of changepoints is large, and pDPA when there are few changepoints.

The focus of this paper is on these pruning techniques, with the aim of trying to combine ideas from PELT and pDPA. This leads to two new algorithms, Functional Pruning Optimal Partitioning (FPOP) and Segment Neighbourhood with Inequality Pruning (SNIP). SNIP uses inequality based pruning to solve the constrained minimisation problem providing an alternative to pDPA which offers greater versatility, especially in the case of multivariate data. FPOP uses functional pruning to solve the penalised minimisation problem efficiently. We show that FPOP always prunes more than PELT. Empirical results suggest that FPOP is efficient for large data sets regardless of the number of changepoints, and we observe that FPOP has a computational cost that is, in some scenarios, even competitive with Binary Segmentation.

The structure of the paper is as follows. We introduce the constrained and penalised optimisation problems for segmenting data in the next section. We then review the existing dynamic programming methods and pruning approaches for solving the penalised optimisation problem in Sect. 3 and for solving the constrained optimisation problem in Sect. 4. The new algorithms, FPOP and SNIP, are developed in Sect. 5, and compared empirically and theoretically with existing pruning methods in Sect. 6. We then evaluate FPOP empirically on both simulated and CNV data in Sect. 7. The paper ends with a discussion.

## Model definition

Assume we have data ordered by time, though the same ideas extend trivially to data ordered by any other attribute such as position along a chromosome. Denote the data by $\mathbf {y}=(y_1,\ldots ,y_n)$. We will use the notation that, for $t\ge s$, the set of observations from time *s* to time *t* is $\mathbf {y}_{s:t}=(y_{s},\ldots ,y_t)$. If we assume that there are *k* changepoints in the data, this will correspond to the data being split into $k+1$ distinct segments. We let the location of the *j*th changepoint be $\tau _j$ for $j=1,\ldots ,k$, and set $\tau _0=0$ and $\tau _{k+1}=n$. The *j*th segment will consist of data points $y_{\tau _{j-1}+1},\ldots ,y_{\tau _j}$. We let $\mathbf {\tau }=(\tau _0,\ldots ,\tau _{k+1})$ be the set of changepoints.

The statistical problem we are considering is how to infer both the number of changepoints and their locations. The specific details of any approach will depend on the type of change, such as change in mean, variance or distribution, that we wish to detect. However a general framework that encompasses many changepoint detection methods is to introduce a cost function for each segment. The cost of a segmentation can then be defined in terms of the sum of the costs across the segments, and we can infer segmentations through minimising the segmentation cost.

Throughout we will let $\mathcal {C}(\mathbf {y}_{s+1:t})$, for $s< t$, denote the cost for a segment consisting of data points $y_{s+1},\ldots ,y_t$. The cost of a segmentation, $\tau _1,\ldots ,\tau _k$ is then

1 $$\begin{aligned} \sum ^k_{j=0}\mathcal {C}(\mathbf {y}_{\tau _j+1:\tau _{j+1}}). \end{aligned}$$

The form of this cost function will depend on the type of change we are wanting to detect. One generic approach to defining these segments is to introduce a model for the data within a segment, and then to let the cost be minus the maximum log-likelihood for the data in that segment. If our model assumes that the data is independent and identically distributed with segment-specific parameter $\mu$ then

2 $$\begin{aligned} \mathcal {C}(\mathbf {y}_{s+1:t})=\min _{\mu }\sum _{i=s+1}^{t}-\log (p(y_i|\mu )). \end{aligned}$$

In this formulation we are detecting changes in the value of the parameter, $\mu$, across segments.

For example if $\mu$ is the mean in Normally distributed data, with known variance $\sigma ^2$, then the cost for a segment would simply be

3 $$\begin{aligned} \mathcal {C}(\mathbf {y}_{s+1:t})= \frac{1}{2\sigma ^2}\sum _{i=s+1}^t\left( y_i-\frac{1}{t-s}\sum _{j=s+1}^t y_j\right) ^2, \end{aligned}$$

which is just a quadratic error loss. We have removed a term that does not depend on the data and is linear in segment length, as this term does not affect the solution to the segmentation problem. The cost for a segment can also include a term that depends on the length of segment. Such a cost appears within a minimum description length criteria Davis et al. (2006), where the cost for a segment $y_{s+1:t}$ would also include a $\log (t-s)$ term.

### Segmenting data using penalised and constrained optimisation

If we know the number of changepoints in the data, *k*, then we can infer their location through minimising (1) over all segmentations with *k* changepoints. Normally however *k* is unknown, and thus has to be estimated. A common approach is to define

4 $$\begin{aligned} C_{k,n}=\min _{\varvec{\tau }}\left[ \sum ^k_{j=0} \mathcal {C}(\mathbf {y}_{\tau _j+1:\tau _{j+1}})\right] , \end{aligned}$$

the minimum cost of a segmenting data $y_{1:n}$ with *k* changepoints. As *k* increases we have more flexibility in our model for the data, therefore $C_{k,n}$ will often be monotonically decreasing in *k* and estimating the number of changepoints by minimising $C_{k,n}$ is not possible. One solution is to solve (4) for a fixed value of *k* which is either assumed to be known or chosen separately. We call this problem the *constrained minimisation problem*.

If *k* is not known, then a common approach is to calculate $C_{k,n}$ and the corresponding segmentations for a range of values, $k=0,1,\ldots ,K$, where *K* is some chosen maximum number. We can then estimate the number of changepoints by minimising $C_{k,n}+f(k,n)$ over *k* for some suitable penalty function *f*(*k*, *n*).

Choosing a good value for *f*(*k*, *n*) is still very much an open problem. The most common choices of *f*(*k*, *n*), for example SIC (Schwarz 1978) and AIC (Akaike 1974) are linear in *k*, however these are only consistent in specific cases and rely on assumptions made about the data generating process which in practice is generally unknown. Recent work in Haynes et al. (2014) looks at picking penalty functions in greater detail, offering ranges of penalties that give good solutions.

If the penalty function is linear in *k*, with $f(k,n)=\beta k$ for some $\beta >0$ (which may depend on *n*), then we can directly find the number of changepoints and corresponding segmentation by noting that

5 $$\begin{aligned} \min _k \left[ C_{k,n}+\beta k\right]= & {} \min _{k,\varvec{\tau }}\left[ \sum ^k_{j=0} \mathcal {C}(\mathbf {y}_{\tau _j+1:\tau _{j+1}})\right] +\beta k, \nonumber \\= & {} \min _{k,\varvec{\tau }}\left[ \sum ^k_{j=0} \mathcal {C}(\mathbf {y}_{\tau _j+1:\tau _{j+1}})+\beta \right] -\beta . \end{aligned}$$

We call the minimisation problem in (5) the *penalised minimisation problem*.

In both the constrained and penalised cases we need to solve a minimisation problem to find the optimal segmentation under our criteria. There are dynamic programming algorithms for solving each of these minimisation problems. For the constrained case this is achieved using the Segment Neighbourhood Search algorithm (see Sect. 4.1), whilst for the penalised case this can be achieved using the Optimal Partitioning algorithm (see Sect. 3.1).

Solving the constrained case offers a way to get segmentations for $k=0,1,\ldots ,K$ changepoints, and thus gives insight into how the segmentation varies with the number of segments. However, a big advantage of the penalised case is that it incorporates model selection into the problem itself, and therefore it is often computationally more efficient when dealing with an unknown value of *k*. In the following we will use the terminology optimal segmentation to define segmentations that are the solution to either the penalised or constrained minimisation problem, with the context making it clear as to which minimisation problem it relates to.

### Conditions for pruning

The focus of this paper is on methods for speeding up these dynamic programming algorithms using pruning methods. The pruning methods can be applied under one of two conditions on the segment costs:

**C1** The cost function satisfies

$$\begin{aligned}&\mathcal {C}(\mathbf {y}_{s+1:t})=\min _{\mu }\sum _{i=s+1}^{t}\gamma (y_i,\mu ), \end{aligned}$$

for some function $\gamma (\cdot ,\cdot )$, with parameter $\mu$.

**C2** There exists a constant $\kappa$ such that for all $s<t<T$,

$$\begin{aligned}&\mathcal {C}(\mathbf {y}_{s+1:t})+\mathcal {C}(\mathbf {y}_{t+1:T})+\kappa \le \mathcal {C}(\mathbf {y}_{s+1:T}). \end{aligned}$$

Condition C1 will be used by functional pruning (which is discussed in Sects. 4.2 and 5.1). Condition C2 will be used by the inequality based pruning (Sects. 3.2 and 5.2).

Note that C1 is a stronger condition than C2. If C1 holds then C2 also holds with $\kappa =0$ and this is true for many practical cost functions. For example it is easily seen that for the negative log-likelihood (2) C1 holds with $\gamma (y_i,\mu )=-\log (p(y_i|\mu ))$ and C2 holds with $\kappa =0$. By comparison, segment costs that are the sum of (2) and a term that depends non-linearly on the length of the segment will obey C2 but not C1.

## Solving the penalised optimisation problem

We first consider solving the penalised optimisation problem (5) using a dynamic programming approach. The initial algorithm, Optimal Partitioning (Jackson et al. 2005), will be discussed first before mentioning how pruning can be used to reduce the computational cost.

### Optimal Partitioning

Consider segmenting the data $\mathbf {y}_{1:t}$. Denote *F*(*t*) to be the minimum value of the penalised cost (5) for segmenting such data, with $F(0)=-\beta$. The idea of Optimal Partitioning is to split the minimisation over segmentations into the minimisation over the position of the last changepoint, and then the minimisation over the earlier changepoints. We can then use the fact that the minimisation over the earlier changepoints will give us the value $F(\tau ^*)$ for some $\tau ^*<t$

$$\begin{aligned} F(t)&=\min _{\varvec{\tau },k}\sum _{j=0}^k\left[ \mathcal {C} (\mathbf {y}_{\tau _j+1:\tau _{j+1}})+\beta \right] -\beta ,\\&=\min _{\varvec{\tau },k}\left\{ \sum _{j=0}^{k-1} \left[ \mathcal {C}(\mathbf {y}_{\tau _j+1:\tau _{j+1}})+\beta \right] +\mathcal {C}(\mathbf {y}_{\tau _k+1:t})+\beta \right\} -\beta ,\\&=\min _{\tau ^*}\left\{ \min _{\varvec{\tau },k'} \sum _{j=0}^{k'}\left[ \mathcal {C}(\mathbf {y}_{\tau _j+1: \tau _{j+1}}) +\beta \right] \right. \\&\left. \quad \qquad \qquad -\beta +\mathcal {C} (\mathbf {y}_{\tau ^*+1:t})+\beta \right\} ,\\&=\min _{\tau ^*}\left\{ F(\tau ^*)+\mathcal {C} (\mathbf {y}_{\tau ^*+1:t})+\beta \right\} . \end{aligned}$$

Hence we obtain a simple recursion for the *F*(*t*) values

6 $$\begin{aligned} F(t)=\min _{0\le \tau <t}\left[ F(\tau ) + \mathcal {C}(\mathbf {y}_{\tau +1:t}) + \beta \right] . \end{aligned}$$

The segmentations themselves can be recovered by first taking the arguments which minimise (6)

7 $$\begin{aligned} \tau ^*_t={\mathop {\hbox {arg min}}\limits _{0\le \tau <t}}\left[ F(\tau ) + \mathcal {C}(\mathbf {y}_{\tau +1:t}) + \beta \right] , \end{aligned}$$

which give the optimal location of the last changepoint in the segmentation of $y_{1:t}$.

If we denote the vector of ordered changepoints in the optimal segmentation of $y_{1:t}$ by *cp*(*t*), with $cp(0)=\emptyset$, then the optimal changepoints up to a time *t* can be calculated recursively

$$\begin{aligned} cp(t)=(cp(\tau ^*_t),\tau ^*_t). \end{aligned}$$

As Eq. (6) is calculated for time steps $t=1,2,\ldots ,n$ and each time step involves a minimisation over $\tau =0,1,\ldots ,t-1$ the computation takes $\mathcal {O}(n^2)$ time.

### PELT

One way to increase the efficiency of Optimal Partitioning is discussed in Killick et al. (2012) where they introduce the PELT (Pruned Exact Linear Time) algorithm. PELT works by limiting the set of potential previous changepoints (i.e. the set over which $\tau$ is chosen in the minimisation in Eq. 6). They show that if condition C2 holds for some $\kappa$, and if

8 $$\begin{aligned} F(s)+\mathcal {C}(\mathbf {y}_{(s+1:t)})+\kappa > F(t), \end{aligned}$$

then at any future time $T>t$, *s* can never be the optimal location of the most recent changepoint prior to *T*.

This means that at every time step *t* the left hand side of Eq. (8) can be calculated for all potential values of the last changepoint. If the inequality holds for any individual *s* then that *s* can be discounted as a potential last changepoint for all future times. Thus the update rules (6) and (7) can be restricted to a reduced set of potential last changepoints, $\tau$, to consider. This set, which we shall denote as $R_t$, can be updated simply by

9 $$\begin{aligned} R_{t+1}=\{\tau \in \{R_{t}\cup \{t\}\}:F(\tau )+\mathcal {C}(\mathbf {y}_{(\tau +1):t})+\kappa \le F(t)\}. \end{aligned}$$

This pruning technique, which we shall refer to as *inequality based pruning*, forms the basis of the PELT method.

Since at each time step in the PELT algorithm the minimisation is being run over fewer values it is expected that this method will be more efficient than the basic Optimal Partitioning algorithm. In Killick et al. (2012) it is shown to be at least as efficient as Optimal Partitioning, with PELT’s computational cost being bounded above by $\mathcal {O}(n^2)$. Under certain conditions the expected computational cost can be shown to be bounded by *Ln* for some constant $L<\infty$. These conditions are given fully in Killick et al. (2012), the most important of which is that the expected number of changepoints in the data increases linearly with the length of the data, *n*.

## Solving the constrained optimisation problem

We now consider applications of dynamic programming to solve the constrained optimisation problem (4). These methods assume a maximum number of changepoints that are to be considered, *K*, and then solve the constrained optimisation problem for all values of $k=1,2,\ldots ,K$. We first describe the initial algorithm, Segment Neighbourhood Search (Auger and Lawrence 1989), and then an approach that uses pruning.

### Segment Neighbourhood Search

Take the constrained case (4) which segments the data up to *t*, for $t\ge k+1$, into $k+1$ segments (using *k* changepoints), and denote the minimum value of the cost by $C_{k,t}$. The idea of Segment Neighbourhood Search is to derive a relationship between $C_{t,k}$ and $C_{s,k-1}$ for $s<t$:

$$\begin{aligned} C_{k,t}&=\min _{\varvec{\tau }}\sum _{j=0}^k\mathcal {C} (\mathbf {y}_{\tau _j+1:\tau _{j+1}}),\\&= \min _{{\tau _k}}\left[ \min _{\varvec{\tau }_{1:k-1}} \sum _{j=0}^{k-1}\mathcal {C}(\mathbf {y}_{\tau _j+1:\tau _{j+1}}) +\mathcal {C}(\mathbf {y}_{\tau _k+1:\tau _{k+1}})\right] ,\\&=\min _{{\tau _k}}\left[ C_{k-1,\tau _k}+\mathcal {C}(\mathbf {y}_{\tau _k+1 :\tau _{k+1}})\right] . \end{aligned}$$

Thus the following recursion is obtained:

10 $$\begin{aligned} C_{k,t}=\min _{\tau \in \{k,\ldots ,t-1\}}\left[ C_{k-1,\tau } +\mathcal {C}(\mathbf {y}_{\tau +1:t})\right] . \end{aligned}$$

If this is run for all values of *t* up to *n* and for $k=2,\ldots ,K$, then the exact segmentations with $1,\ldots ,K$ segments can be acquired.

To extract the exact segmentation we first let $\tau ^*_l(t)$ denote the optimal position of the last changepoint if we segment data $\mathbf {y}_{1:t}$ using *l* changepoints. This can be calculated asThen if we let $(\tau _1^k,\ldots ,\tau _k^k)$ be the set of changepoints in the segmentation of $\mathbf {y}_{1:n}$ into $k+1$ segments, we have $\tau _k^k=\tau ^*_k(n)$. Furthermore we can calculate the other changepoint positions recursively for $l=k-1,\ldots ,1$ using

$$\begin{aligned} \tau _l^k(n)=\tau ^*_l(\tau _{l+1}^k). \end{aligned}$$

For a fixed value of *k* Eq. (10) is computed for $t\in 1,\ldots ,n$. Then for each *t* the minimisation is done for $\tau =1,\ldots ,t-1$. This means that $\mathcal {O}(n^2)$ calculations are needed. However, to also identify the optimal number of changepoints this then needs to be done for $k\in 1,\ldots ,K$ so the total computational cost in time can be seen to be $\mathcal {O}(Kn^2)$.

### Pruned Segment Neighbourhood Search

Rigaill (2010) has developed techniques to increase the efficiency of Segment Neighbourhood Search using functional pruning. These form the basis of a method called pruned Dynamic Programming Algorithm (pDPA). A more generic implementation of this method is presented in Cleynen et al. (2012). Here we describe how this algorithm can be used to calculate the $C_{k,t}$ values. Once these are calculated, the exact segmentation can be extracted as in Segment Neighbourhood Search.

Assuming condition C1, the segment cost function can be split into the component parts $\gamma (y_i,\mu )$, which depend on the parameter $\mu$. We can then define new cost functions, $Cost^{\tau }_{k,t}(\mu )$, as the minimal cost of segmenting data $y_{1:t}$ into *k* segments, with a most recent changepoint at $\tau$, and where the segment after $\tau$ is conditioned to have parameter $\mu$. Thus for $\tau \le t-1$,

11 $$\begin{aligned} Cost^\tau _{k,t}(\mu )=C_{k-1,\tau }+\sum _{i=\tau +1}^t \gamma (y_i,\mu ), \end{aligned}$$

and $$Cost^t_{k,t}(\mu )=C_{k-1,t}$$.

These functions, which are stored for each candidate changepoint, can then be updated at each new time step as for $\tau \le t-1$

12 $$\begin{aligned} Cost^\tau _{k,t}(\mu )=Cost^\tau _{k,t-1}(\mu )+\gamma (y_{t},\mu ). \end{aligned}$$

By taking the minimum of $Cost^\tau _{k,t}(\mu )$ over $\mu$, the individual terms of the right hand side of Eq. (10) can be recovered. Therefore, by further minimising over $\tau$, the minimum cost $C_{k,t}$ can be returned

$$\begin{aligned} \min _\tau \min _\mu Cost^\tau _{k,t}(\mu )&= \min _\tau \min _\mu \left[ C_{k-1,\tau }+\sum _{i=\tau +1}^t\gamma (y_i,\mu )\right] ,\\&=\min _\tau \left[ C_{k-1,\tau }+\min _\mu \sum _{i=\tau +1}^t\gamma (y_i,\mu )\right] ,\\&=\min _\tau \left[ C_{k-1,\tau }+\mathcal {C}(\mathbf {y}_{\tau +1:t})\right] ,\\&= C_{k,t}. \end{aligned}$$

By interchanging the order of minimisation the values of the potential last changepoint, $\tau$, can be pruned whilst allowing for changes in $\mu$. First we define the function $Cost^*_{k,t}(\mu )$ as follows

$$\begin{aligned} Cost^*_{k,t}(\mu )= \min _{\tau }Cost^\tau _{k,t}(\mu ). \end{aligned}$$

We can now get a recursion for $Cost^*_{k,t}(\mu )$ by splitting the minimisation over the most recent changepoint $\tau$ into the two cases $\tau \le t-1$ and $\tau =t$

$$\begin{aligned} Cost^*_{k,t}(\mu )= & {} \min \left\{ \min _{\tau \le t-1} Cost^\tau _{k,t}(\mu ), Cost^t_{k,t}(\mu ) \right\} ,\\= & {} \min \left\{ \min _{\tau \le t-1} Cost^\tau _{k,t-1}(\mu )\right. \\&\qquad \quad \left. +\gamma (y_t,\mu ) , C_{k-1,t} \right\} , \end{aligned}$$

which gives

$$\begin{aligned} Cost^*_{k,t}(\mu )=\min \left\{ Cost^*_{k,t-1}(\mu )+\gamma (y_t,\mu ), C_{k-1,t} \right\} . \end{aligned}$$

The idea of pDPA is to use this recursion for $Cost^*_{k,t}(\mu )$. We can then use the fact that $C_{k,t}=\min _\mu Cost^*_{k,t}(\mu )$ to calculate the $C_{k,t}$ values. In order to do this we need to be able to represent this function of $\mu$ in an efficient way. This can be done if $\mu$ is a scalar, because for any value of $\mu$, $Cost^*_{k,t}(\mu )$ is equal to the value of $Cost^\tau _{k,t}(\mu )$ for some value of $\tau$. Thus we can partition the possible values of $\mu$ into intervals, with each interval corresponding to a value for $\tau$ for which $Cost^*_{k,t}(\mu )=Cost^\tau _{k,t}(\mu )$.

**Fig. 1:**
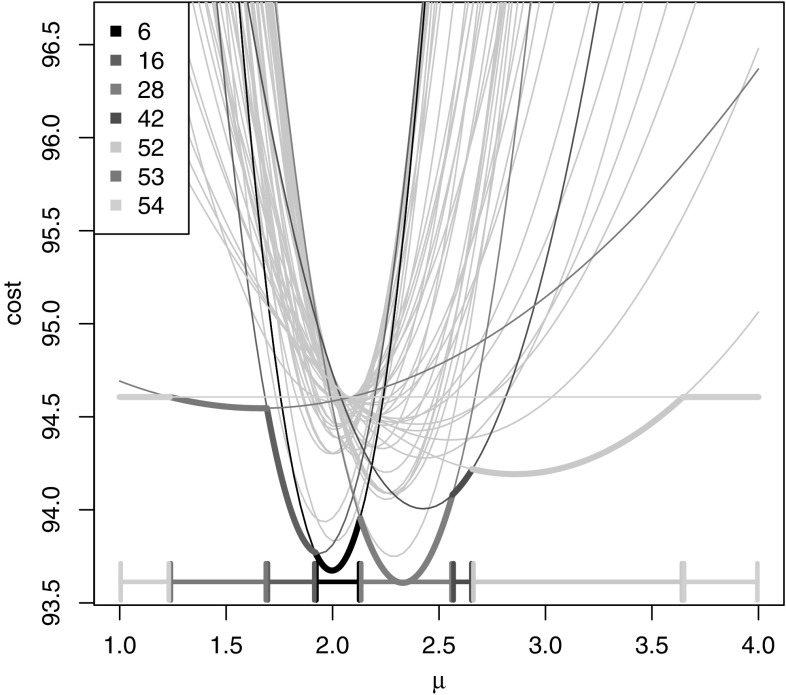
Cost functions, $Cost_{k,\tau }(\mu ,t)$ for $\tau =0,\ldots ,54$ and $t=54$ and the corresponding $C^*_{k}(\mu ,t)$ (*in bold*) for a change in mean using the negative normal log-likelihood cost function (3). *Coloured lines* correspond to $Cost_{k,\tau }(\mu ,t)$ that contribute to $C^*_{k}(\mu ,t)$, with the *coloured horizontal lines* showing the intervals of $\mu$ for which each value of $\tau$ is such that $Cost_{k,\tau }(\mu ,t)=C^*_{k}(\mu ,t)$. *Faded lines* correspond to candidates which have previously been pruned, and do not contribute to $C^*_{k}(\mu ,t)$

To make the idea concrete, an example of $Cost^*_{k,t}(\mu )$ is given in Fig. 1 for a change in mean using the cost function given in (3). As each $\gamma (y_i,\mu )$ is quadratic in $\mu$ then the sum of these, $Cost^\tau _{k,t}(\mu )$, is also a quadratic function in this case. In this example there are 8 intervals of $\mu$ corresponding to 7 different values of $\tau$ for which $Cost^*_{k,t}(\mu )=Cost^\tau _{k,t}(\mu )$. The pDPA algorithm needs to just store the 7 different $Cost^\tau _{k,t}(\mu )$ functions, and the corresponding sets.

**Fig. 2:**
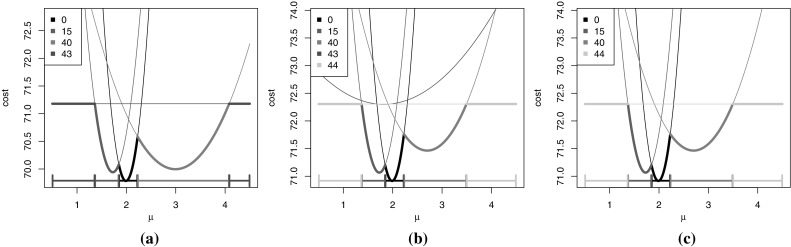
Example of the pDPA algorithm over two time-steps. On each plot we show individual $Cost^\tau _{k,t}(\mu )$ functions that are stored, together with the intervals (*along the bottom*) for which each candidate last changepoint is optimal. In *bold* is the value of $Cost^*_{k,t}(\mu )$. For this example $t=43$ and we are detecting a change in mean (see Sect. 2). (**a**) 4 candidates are optimal for some interval of $\mu$, however at $t=44$ (**b**), when the candidate functions are updated and the new candidate is added, then the candidate $\tau =43$ is no longer optimal for any $\mu$ and hence can be pruned (**c**)

Formally speaking we define the set of intervals for which $Cost^*_{k,t}(\mu )=Cost^\tau _{k,t}(\mu )$ as $Set_{k,t}^\tau$. The recursion for $Cost^*_{k,t}(\mu )$ can be used to induce a recursion for these sets. First define:

13 $$\begin{aligned} I^\tau _{k,t}=\{\mu : Cost^\tau _{k,t}(\mu )\le C_{k-1,t}\}. \end{aligned}$$

Then, for $\tau \le t-1$ we have

$$\begin{aligned} Set_{k,t}^{\tau }= & {} \left\{ \mu :Cost^\tau _{k,t}(\mu )= Cost^*_{k,t}(\mu )\right\} ,\\= & {} \left\{ \mu :Cost^\tau _{k,t-1}(\mu )+\gamma (y_t,\mu )\right. \\&\quad = \left. \min \left\{ Cost^*_{k,t-1}(\mu )+\gamma (y_t,\mu ),C_{k-1,t} \right\} \right\} . \end{aligned}$$

Remembering that $Cost^\tau _{k,t-1}(\mu )+\gamma (y_t,\mu )\ge Cost^*_{k,t-1}(\mu )+\gamma (y_t,\mu )$, we have that for $\mu$ to be in $Set_{k,t}^{\tau }$ we need that $Cost^\tau _{k,t-1}(\mu )=Cost^*_{k,t-1}(\mu )$, and that $Cost^\tau _{k,t-1}(\mu )+\gamma (y_t,\mu )\le C_{k-1,t}$. The former condition corresponds to $\mu$ being in $Set_{k,t-1}^{\tau }$ and the second that $\mu$ is in $I^\tau _{k,t}$. So for $\tau \le t-1$

$$\begin{aligned} Set^{\tau }_{k,t}=Set^{\tau }_{k,t-1} \cap I^\tau _{k,t}. \end{aligned}$$

If this $Set^{\tau }_{k,t}=\emptyset$ then the value $\tau$ can be pruned, as $Set^{\tau }_{k,T}=\emptyset$ for all $T>t$.

If we denote the range of values $\mu$ can take to be *D*, then we further have that

$$\begin{aligned} Set^{t}_{k,t}=D \backslash \left[ \displaystyle \bigcup _{\tau }I^\tau _{k,t}\right] , \end{aligned}$$

where *t* can be pruned straight away if $Set^t_{k,t}=\emptyset$.

An example of the pDPA recursion is given in Fig. 2 for a change in mean using the negative normal log-likelihood cost function (3). The left-hand plot shows $Cost^*_{k,t}(\mu )$. In this example there are 5 intervals of $\mu$ corresponding to 4 different values of $\tau$ for which $Cost^*_{k,t}(\mu )=Cost^\tau _{k,t}(\mu )$. When we analyse the next data point, we update each of these four $Cost^\tau _{k,t}(\mu )$ functions, using $Cost^\tau _{k,t+1}(\mu )=Cost^\tau _{k,t}(\mu )+\gamma (y_{t+1},\mu )$, and introduce a new curve corresponding to a change-point at time $t+1$, $Cost^{t+1}_{k,t+1}(\mu )=C_{k-1,t+1}$ (see middle plot). We can then prune the functions which are no longer optimal for any $\mu$ values, and in this case we remove one such function (see right-hand plot).

pDPA can be shown to be bounded in time by $\mathcal {O}(Kn^2)$. Rigaill (2010) further analyse the time complexity of pDPA and show it empirically to be $\mathcal {O}(Kn\log n)$, further indications towards this will be presented in Sect. 7. However pDPA has a computational overhead relative to Segment Neighbourhood Search, as it requires calculating and storing the $Cost^\tau _{k,t}(\mu )$ functions and the corresponding sets $Set_{k,t}^\tau$. Currently implementations of pDPA have only been possible for models with scalar segment parameters $\mu$, due to the difficulty of calculating the sets in higher dimensions. Being able to efficiently store and update the $Cost^\tau _{k,t}(\mu )$ has also restricted applications primarily to models where $\gamma (y,\mu )$ corresponds to the log-likelihood of an exponential family. However this still includes a wide-range of changepoint applications, including that of detecting CNVs that we consider in Sect. 7. The cost of updating the sets depends heavily on whether the updates (13) can be calculated analytically, or whether they require the use of numerical methods.

## New changepoint algorithms

Two natural ways of extending the two methods introduced above will be examined in this section. These are, respectively, to apply functional pruning (Sect. 4.2) to Optimal Partitioning, and to apply inequality based pruning (Sect. 3.2) to Segment Neighbourhood Search. These lead to two new algorithms, which we call Functional Pruning Optimal Partitioning (FPOP) and Segment Neighbourhood with Inequality Pruning (SNIP).

### Functional Pruning Optimal Partitioning

Functional Pruning Optimal partitioning (FPOP) provides a version of Optimal Partitioning (Jackson et al. 2005) which utilises functional pruning to increase the efficiency. As will be discussed in Sect. 6 and shown in Sect. 7, FPOP provides an alternative to PELT which is more efficient in certain scenarios. The approach used by FPOP is similar to the approach for pDPA in Sect. 4.2, however the theory is slightly simpler here as there is no longer the need to condition on the number of changepoints.

We assume condition C1 holds, that the cost function, $\mathcal {C}(\mathbf {y}_{\tau +1:t})$, can be split into component parts $\gamma (y_i,\mu )$ which depend on the parameter $\mu$. Cost functions $Cost_t^\tau$ can then be defined as the minimal cost of the data up to time *t*, conditional on the last changepoint being at $\tau$ and the last segment having parameter $\mu$. Thus for $\tau \le t-1$

14 $$\begin{aligned} Cost_t^\tau (\mu )=F(\tau )+\beta +\sum _{i=\tau +1}^t\gamma (y_i,\mu ), \end{aligned}$$

   and $$Cost_t^t(\mu )=F(t)+\beta $$.

These functions, which only need to be stored for each candidate changepoint, can then be recursively updated at each time step, $\tau \le t-1$

15 $$\begin{aligned} Cost^\tau _{t}(\mu )=Cost^\tau _{t-1}(\mu )+\gamma (y_{t},\mu ). \end{aligned}$$

Given the cost functions $Cost^\tau _t(\mu )$ the minimal cost *F*(*t*) can be returned by minimising over both $\tau$ and $\mu$:

$$\begin{aligned} \min _\tau \min _\mu Cost_t^\tau (\mu )&= \min _\tau \min _\mu \left[ F(\tau )+\beta +\sum _{i=\tau +1}^t\gamma (y_i,\mu )\right] ,\\&=\min _\tau \left[ F(\tau )+\beta +\min _\mu \sum _{i=\tau +1}^t\gamma (y_i,\mu )\right] ,\\&=\min _\tau \left[ F(\tau )+\beta +\mathcal {C}(\mathbf {y}_{\tau +1:t})\right] ,\\&=F(t). \end{aligned}$$

As before, by interchanging the order of minimisation, the values of the potential last changepoint, $\tau$, can be pruned whilst allowing for a varying $\mu$. Firstly we will define the function $Cost_{t}^*(\mu )$, the minimal cost of segmenting data $y_{1:t}$ conditional on the last segment having parameter $\mu$

$$\begin{aligned} Cost^*_t(\mu )=\min _{\tau } Cost^\tau _t(\mu ). \end{aligned}$$

Note that if a potential last changepoint $\tau _1$ doesn’t form part of the piecewise function $Cost^*_t(\mu )$ for a time *t* (i.e. there doesn’t exist $\mu$ such that $Cost^*_t(\mu )= Cost^{\tau _1}_t(\mu )$), then this implies that for any given $\mu$ we can find $\tau _2$ such that $Cost^{\tau _2}_t(\mu )<Cost^{\tau _1}_t(\mu )$ and further, from the recursion given in (15), $Cost^{\tau _2}_T(\mu )<Cost^{\tau _1}_T(\mu )$ for all $T>t$. Hence if $\tau _1$ doesn’t form part of the piecewise function $Cost^*_t(\mu )$ at time *t* then it can be pruned from all future time steps.

We will update these functions recursively over time, and use $F(t)=\min _\mu Cost^*_t(\mu )$ to then obtain the solution of the penalised minimisation problem. The recursions for $Cost^*_t(\mu )$ are obtained by splitting the minimisation over $\tau$ into $\tau \le t-1$ and $\tau =t$

$$\begin{aligned}&Cost^*_t(\mu )=\min \left\{ \min _{\tau \le t-1} Cost^\tau _t(\mu ) , Cost^t_t(\mu )\right\} ,\\&\quad =\min \left\{ \min _{\tau \le t-1} Cost^\tau _{t-1}(\mu )+\gamma (y_t,\mu ) , Cost^t_t(\mu )\right\} , \end{aligned}$$

which then gives

$$\begin{aligned} Cost^*_t(\mu )=\min \{ Cost^*_{t-1}(\mu )+\gamma (y_t,\mu ) , F(t)+\beta \}. \end{aligned}$$

To implement this recursion we need to be able to efficiently store and update $Cost^*_t(\mu )$. As before we do this by partitioning the space of possible $\mu$ values, *D*, into sets where each set corresponds to a value $\tau$ for which $Cost^*_t(\mu )=Cost^\tau _t(\mu )$. We then need to be able to update these sets, and store $Cost^\tau _t(\mu )$ just for each $\tau$ for which the corresponding set is non-empty.

This can be achieved by first defining

16 $$\begin{aligned} I^\tau _{t}=\{\mu : Cost^\tau _{t}(\mu )\le F(t) +\beta \}. \end{aligned}$$

Then, for $\tau \le t-1$, we define

$$\begin{aligned} Set_t^\tau&= \{\mu : Cost_t^\tau (\mu )=Cost_t^*(\mu )\},\\&=\{\mu : Cost_{t-1}^\tau (\mu )+\gamma (y_t,\mu )\\&\qquad =\min {\{Cost_{t-1}^*(\mu )+\gamma (y_t,\mu ) , F(t)+\beta \}}\}. \end{aligned}$$

Remembering that $Cost^\tau _{t-1}(\mu )+\gamma (y_t,\mu )\ge Cost^*_{t-1}(\mu )+\gamma (y_t,\mu )$; we have that for $\mu$ to be in $Set_{t}^{\tau }$ we need that $Cost^\tau _{t-1}(\mu )=Cost^*_{t-1}(\mu )$, and that $Cost^\tau _{t-1}(\mu )+\gamma (y_t,\mu )\le F(t)+\beta$. The former condition corresponds to $\mu$ being in $Set_{t-1}^{\tau }$ and the second that $\mu$ is in $I^\tau _{t}$, so for $\tau \le t-1$

$$\begin{aligned} Set^{\tau }_{t}=Set^{\tau }_{t-1} \cap I^\tau _{t}. \end{aligned}$$

If $Set^{\tau }_{t}=\emptyset$ then the value $\tau$ can be pruned, as then $Set^{\tau }_{T}=\emptyset$ for all $T>t$.

If we denote the range of values $\mu$ can take to be *D*, then we further have that

$$\begin{aligned} Set^{t}_{t}=D \backslash \left[ \displaystyle \bigcup _{\tau }I^\tau _{t}\right] , \end{aligned}$$

where *t* can be pruned straight away if $Set_t^t=\emptyset$.

**Fig. 3:**
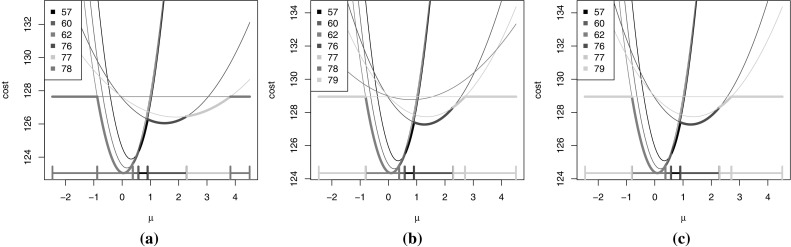
Candidate functions over two time steps, the intervals shown *along the bottom* correspond to the intervals of $\mu$ for which each candidate last changepoint is optimal. When $t=78$ (**a**) 4 candidates are optimal for some interval of $\mu$, however at $t=79$ (**b**), when the candidate functions are updated and the new candidate is added, then candidate $\tau =78$ is no longer optimal for any $\mu$ and hence can be pruned (**c**)

This updating of the candidate functions and sets is illustrated in Fig. 3 where the *Cost* functions and *Set* intervals are displayed across two time steps. In this example a change in mean has been considered, using the negative normal log-likelihood cost function (3). As each $\gamma (y_i,\mu )$ is quadratic in $\mu$ then the sum of these, $Cost^\tau _{k,t}(\mu )$, is also a quadratic function in this case. The bold line on the left-hand graph corresponds to the function $Cost^*_t(\mu )$ and is made up of 7 pieces which relate to 6 candidate last changepoints. As the next time point is analysed the six $Cost_t^\tau (\mu )$ functions are updated using the formula $Cost_{t+1}^\tau (\mu )=Cost_t^\tau (\mu )+\gamma (y_{t+1},\mu )$ and a new function, $Cost_{t+1}^{t+1}(\mu )=F(t+1)+\beta$, is introduced corresponding to placing a changepoint at time $t+1$ (see middle plot). The functions which are no longer optimal for any values of $\mu$ (i.e. do not form any part of $Cost^*_{t+1}(\mu )$) can then be pruned, and one such function is removed in the right-hand plot.

Once again we denote the set of potential last changes to consider as $R_{t}$ and then restrict the update rules (6) and (7) to $\tau \in R_{t}$. This set can then be recursively updated at each time step

17 $$\begin{aligned} R_{t+1} = \{ \tau \in \{R_{t} \cup \{t\}\} : Set_{t}^\tau \ne \emptyset \}. \end{aligned}$$

These steps can then be applied directly to an Optimal Partitioning algorithm to form the FPOP method and the full pseudocode for this is presented in Algorithm 1.
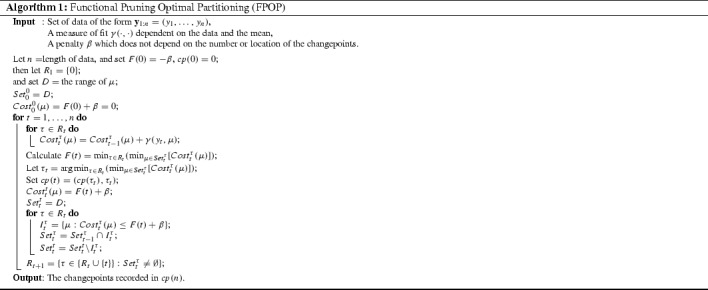


### Segment Neighbourhood with Inequality Pruning

In a similar vein to Sect. 5.1, Segment Neighbourhood Search can also benefit from using pruning methods. In Sect. 4.2 the method pDPA was discussed as a fast pruned version of Segment Neighbourhood Search. In this section a new method, Segment Neighbourhood with Inequality Pruning (SNIP), will be introduced. This takes the Segment Neighbourhood Search algorithm and uses inequality based pruning to increase the speed.

Under condition (C2) the following result can be proved for Segment Neighbourhood Search and this will enable points to be pruned from the candidate changepoint set.

#### **Theorem 1**

Assume that there exists a constant, $\kappa$, such that condition C2 holds. If, for any $k\ge 1$ and $s<t$

18 $$\begin{aligned} C_{k-1,s}+\mathcal {C}(\mathbf {y}_{s+1:t})+\kappa > C_{k-1,t}, \end{aligned}$$

then at any future time $T>t$, *s* cannot be the position of the last changepoint in the exact segmentation of $y_{1:T}$ with *k* changepoints.

#### *Proof*

The idea of the proof is to show that a segmentation of $y_{1:T}$ into *k* segments with the last changepoint at *t* will be better than one with the last changepoint at *s* for all $T>t$.

Assume that (18) is true. Now for any $t<T\le n$

$$\begin{aligned}&C_{k-1,s}+\mathcal {C}(\mathbf {y}_{s+1:t})+\kappa +>C_{k-1,t},\\&\quad C_{k-1,s}+\mathcal {C}(\mathbf {y}_{s+1:t}) +\kappa +\mathcal {C} (\mathbf {y}_{t+1:T})\\&\qquad >C_{k-1,t}+\mathcal {C}(\mathbf {y}_{t+1:T}),\\&\quad C_{k-1,s}+\mathcal {C}(\mathbf {y}_{s+1,T})>C_{k-1,t} +\mathcal {C}(\mathbf {y}_{t+1,T}),\quad \text{(by } \text{ C2). } \end{aligned}$$

Therefore for any $T>t$ the cost $C_{k-1,s}+\mathcal {C}(\mathbf {y}_{s+1,T})>C_{k,T}$ and hence *s* cannot be the optimal location of the last changepoint when segmenting $\mathbf {y}_{1:T}$ with *k* changepoints. $\square$

Theorem 1 implies that the update rule (10) can be restricted to a reduced set over $\tau$ of potential last changes to consider without losing the exactness of Segment Neighbourhood Search. This set, which we shall denote as $R_{k,t}$, can be updated simply by

19 $$\begin{aligned} R_{k,t+1}&=\{v\in \{R_{k,t}\cup \{t\}\}:C_{k-1,v} +\mathcal {C}(\mathbf {y}_{v+1,t})+\kappa < C_{k-1,t}\}. \end{aligned}$$

This new algorithm, SNIP, is described fully in Algorithm 2.
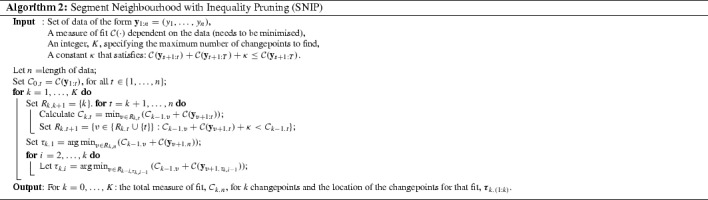

**Fig. 4:**
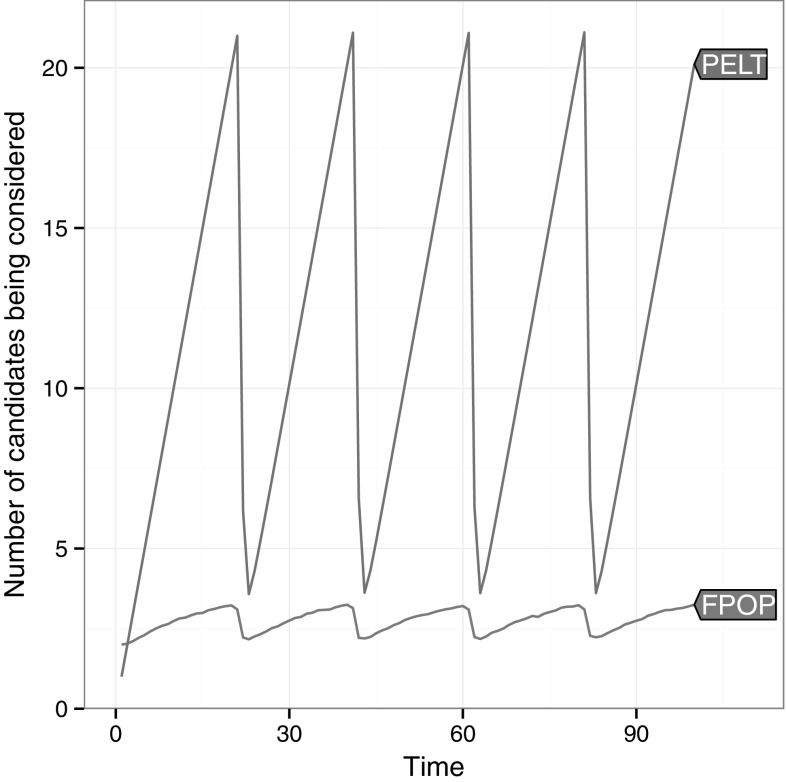
Comparison of the number of candidate changepoints stored over time by FPOP and PELT. Averaged over 1000 data sets with changepoints at $t=20,40,60$ and 80

## Comparisons between pruning methods

Functional and inequality based pruning both offer increases in the efficiency in solving both the penalised and constrained problems, however their use depends on the assumptions which can be made on the cost function. Inequality based pruning is dependent on the assumption C2, while functional pruning requires the slightly stronger condition C1.

Functional pruning also requires a larger computational overhead than inequality based pruning. This arises due to the potential difficulties in calculating $Set_t^{\tau }$ for all $\tau$ at a given timepoint *t*. If this calculation can be done efficiently (ie. for a univariate parameter from a model in the exponential family, where the intervals can be calculated analytically) then the algorithm (such as FPOP or pDPA) will be efficient too. In particular, this is infeasible (at least using current approaches) for multi-dimensional parameters, as in this case the intervals $Set_t^{\tau }$ are also multi-dimensional.

If we consider models for which both pruning methods can be implemented, we can compare the extent to which the methods prune. This will give some insight into when the different pruning methods would be expected to work well.

**Fig. 5:**
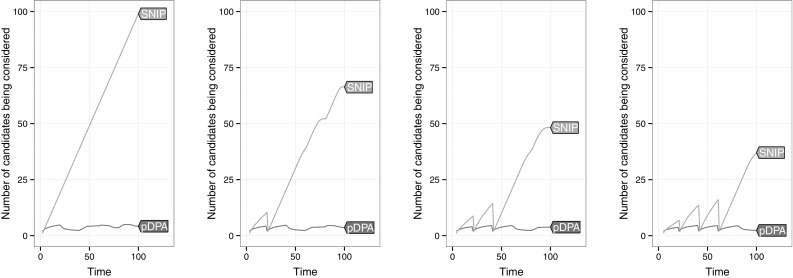
Comparison of the number of candidate changepoints stored over time by pDPA and SNIP at multiple values of *k* in the algorithms (going from left to right $k=2,3,4,5$). Averaged over 1000 data sets with changepoints at $t=20,40,60$ and 80

To explore this in Figs. 4 and 5 we look at the amount of candidates stored by functional and inequality based pruning in each of the two optimisation problems.

As Fig. 4 illustrates, PELT prunes very rarely; only when evidence of a change is particularly high. In contrast, FPOP prunes more frequently keeping the candidate set small throughout. Figure 5 shows similar results for the constrained problem. While pDPA constantly prunes, SNIP only prunes sporadically. In addition SNIP fails to prune much at all for low values of *k*.

Figures 4 and 5 give strong empirical evidence that functional pruning prunes more points than the inequality based method. In fact it can be shown that any point pruned by inequality based pruning will also be pruned at the same time step by functional pruning. This result holds for both the penalised and constrained case and is stated formally in Theorem 2.

### **Theorem 2**

Let $\mathcal {C}(\cdot )$ be a cost function that satisfies condition C1, and consider solving either the constrained or penalised optimisation problem using dynamic programming and either inequality or functional pruning.

Any point pruned by inequality based pruning at time *t* will also have been pruned by functional pruning at the same time.

### *Proof*

We prove this for pruning of optimal partitioning, with the ideas extending directly to the pruning of the Segment Neighbourhood algorithm.

For a cost function which can be decomposed into pointwise costs, it’s clear that condition C2 holds when $\kappa =0$ and hence inequality based pruning can be used. Recall that the point $\tau$ (where $\tau <t$, the current time point) is pruned by inequality based pruning in the penalised case if

$$\begin{aligned} F(\tau )+\mathcal {C}(\mathbf {y}_{\tau +1:t})\ge F(t), \end{aligned}$$

Then, by letting $\hat{\mu }_{\tau }$ be the value of $\mu$ such that $Cost^\tau _t(\mu )$ is minimised, this is equivalent to

$$\begin{aligned} Cost^\tau _t(\hat{\mu }_{\tau })-\beta \ge F(t), \end{aligned}$$

Which can be generalised for all $\mu$ to

$$\begin{aligned} Cost^\tau _t(\mu )\ge F(t)+\beta . \end{aligned}$$

Therefore Eq. (16) holds for no value of $\mu$ and hence $I^\tau _t=\emptyset$ and furthermore $Set^\tau _{t+1}=Set^\tau _t\cap I^\tau _t=\emptyset$ meaning that $\tau$ is pruned under functional pruning.

## Empirical evaluation of FPOP

As explained in Sect. 6 functional pruning leads to a better pruning in the following sense: any point pruned by inequality based pruning will also be pruned by functional pruning. However, functional pruning is computationally more demanding than inequality based pruning. We thus decided to empirically compare the performance of FPOP to PELT (Killick et al. 2012), pDPA (Rigaill 2010), Binary Segmentation (BinSeg), Wild Binary Segmentation (WBS) (Fryzlewicz 2012) and SMUCE (Frick et al. 2014).

PELT and pDPA have been discussed in Sects. 3.2 and 4.2 respectively. Binary Segmentation (Scott and Knott 1974) involves the entire data being scanned for a single changepoint and then splitting into two segments around this change. The process is then repeated on these two segments. This recursion is repeated until a certain criterion is satisfied. Wild Binary Segmentation (Fryzlewicz 2012) takes this method further, taking a randomly drawn number of subsamples from the data and searching these subsamples for a changepoint. As before the data is then split around the changepoint and the process repeated on the two created segments. Lastly SMUCE (Simultaneous Multiscale Changepoint Inference) (Frick et al. 2014) uses a multiscale test at level $\alpha$ and estimates a step function that minimises the number of changepoints whilst lying in the acceptance region of this test.

To do the analysis, we implement FPOP for the quadratic loss (3) in C++, the code for this can be found in the opfp project repository on R-Forge:

https://r-forge.r-project.org/R/?group_id=1851. We assess the runtimes of FPOP on both real microarray data as well as synthetic data. All algorithms were implemented in C++.

**Fig. 6:**
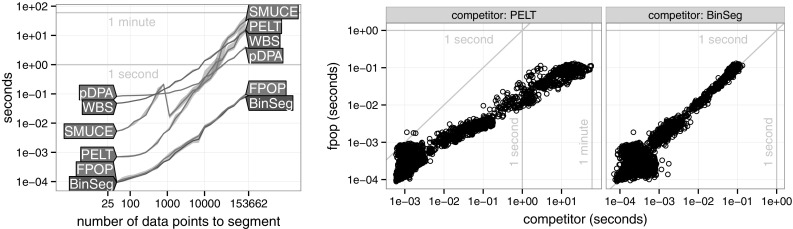
Timings on the tumor micro array benchmark. *Left* Runtimes as a function of the length *n* of the profile (median line and quartile error band). *Middle* Runtimes of PELT and FPOP for the same profiles. *Right* Runtimes of BinSeg and FPOP for the same profiles

### Speed benchmark: 4467 chromosomes from tumour microarrays

Hocking et al. (2014) proposed to benchmark the speed of segmentation algorithms on a database of 4467 problems of size varying from $n=25$ to 153662 data points. These data come from different microarrays data sets (Affymetrix, Nimblegen, BAC/PAC) and different tumour types (leukaemia, lymphoma, neuroblastoma, medulloblastoma).

We compared FPOP to several other segmentation algorithms: pDPA (Rigaill 2010), PELT (Killick et al. 2012), Binary Segmentation (BinSeg), Wild Binary Segmentation (WBS; Fryzlewicz 2012), and SMUCE (Frick et al. 2014). We ran pDPA and BinSeg with a maximum number of changes $K=52$, WBS and SMUCE with default settings, and PELT and FPOP with the SIC penalty.

We used the R microbenchmark package to measure the execution time on each of the 4467 segmentation problems. The R source code for these timings is in benchmark/systemtime.arrays.R in the opfp project repository on R-Forge: https://r-forge.r-project.org/R/?group_id=1851.

Figure 6 shows that the speed of FPOP is comparable to BinSeg, and faster than the other algorithms. As expected, it is clear that the asymptotic behavior of FPOP is similar to pDPA for a large number of data points to segments. Note that for analysing a single data set, WBS could be more easily implemented in parallelised computing environment that the other methods. If done so this would lead to some reduction in it computational cost per data set. For analysing multiple data sets, as here, all methods are trivially parallelisable through analysing each data set on a different CPU.

### Speed benchmark: simulated data with different number of changes

The speed of PELT, BinSeg and pDPA depends on the underlying number of changes. For pDPA and BinSeg the relationship is clear; to cope with a larger number of changes, one needs to increase the maximum number of changes *K*. For a signal of fixed size *n*, the time complexity is expected to be $\mathcal {O}(\log K)$ for BinSeg and $\mathcal {O}(K)$ for pDPA (Rigaill 2010).

**Fig. 7:**
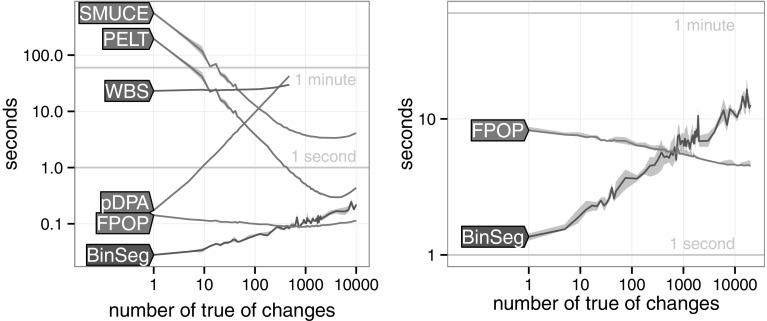
Runtimes in simulated data sets with a variable number of true changepoints (*median line* and quartile error band). *Left* All algorithms in data of size $n=2 \times 10^5$. *Right* BinSeg and FPOP in data of size $n=10^7$

For PELT the expected time complexity is not as clear, but pruning should be more efficient if there are many changepoints. Hence for a signal of fixed size *n*, we expect the runtime of PELT to decrease with the underlying number of changes.

Based on Sect. 6, we expect FPOP to be faster than PELT and pDPA. Thus it seems reasonable to expect FPOP to faster for the whole range of *K*. This is what we empirically check in this section.

To do that we simulated a Gaussian signal with $n=2\times 10^5$ data points, and varied the number of changes *K*. We then repeat the same experiment for signals with $n=10^7$ and timed FPOP and BinSeg only. The R source code for these timings is in benchmark/systemtime.simula tion.R in the opfp project repository on R-Forge: https://r-forge.r-project.org/R/?group_id=1851.

It can be seen in Fig. 7 that FPOP is always faster than pDPA, PELT, WBS, and SMUCE. Interestingly for both $n=2\times 10^5$ and $n=10^7$, FPOP is faster than BinSeg for a true number of changepoints larger than $K=500$.

### Accuracy benchmark: the neuroblastoma data set

Hocking et al. (2013) proposed the neuroblastoma tumor microarray data set for benchmarking changepoint detection accuracy of segmentation models. These data consist of annotated region labels defined by expert doctors when they visually inspected scatterplots of the data. There are 2845 negative labels where there should be no changes (a false positive occurs if an algorithm predicts a change), and 573 positive labels where there should be at least one change (a false negative occurs if an algorithm predicts no changes). There are 575 copy number microarrays, and a total of 3418 labeled chromosomes (separate segmentation problems).

Let *m* be the number of segmentation problems in the train set, let $n_1, \dots , n_m$ be the number of data points to segment in each problem, and let $\mathbf y^{1}\in \mathbb {R}^{n_1}, \dots , \mathbf y^{m}\in \mathbb {R}^{n_m}$ be the vectors of noisy data to segment. Both PELT and pDPA have been applied to this benchmark by first defining a penalty value of $\beta = \lambda n_i$ in (5) for all problems $i\in \{1, \dots , m\}$, and then choosing the constant $\lambda \in \{10^{-8}, \dots , 10^1\}$ that minimises the number of incorrect labels in the train set. To apply this model selection criterion to WBS and SMUCE, we first computed a sequence of models with up to $K=20$ segments (for WBS we used the changepoints.sbs function, and for SMUCE we varied the q parameter).

**Fig. 8:**
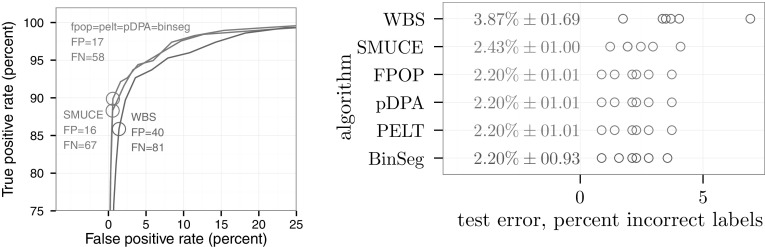
Accuracy results on the neuroblastoma data set. *Left* Train error ROC curves computed by varying the penalty $\lambda$ on the entire data set. *Circles and text* indicate the penalty $\lambda$ which minimized the number of incorrect labels (*FP* false positive, *FN* false negative). *Right* Test error (*circles* 6 test folds; *text* mean and standard deviation)

First, we computed train error ROC curves by considering the entire database as a train set, and computing false positive and true positive rates for each penalty $\lambda$ parameter (Fig. 8, left). The ROC curves suggest that FPOP, PELT, pDPA, and BinSeg have the best detection accuracy, followed by SMUCE, and then WBS.

Second, we performed cross-validation to estimate the test error of each algorithm. We divided the labeled segmentation problems into six folds. For each fold we designate it as a test set, and use the other five folds as a train set. For each algorithm we used grid search to choose the penalty $\lambda$ parameter which had the minimum number of incorrect labels in the train set. We then count the number of incorrect labels on the test set. In agreement with the ROC curves, FPOP/pDPA/PELT/BinSeg had the smallest test error (2.2 %), followed by SMUCE (2.43 %), and then WBS (3.87 %). Using a paired one-sided $t_5$-test, FPOP had significantly less test error than WBS ($p=0.005$) but not SMUCE ($p=0.061$).

### Accuracy on the WBS simulation benchmark

We assessed the performance of FPOP using the simulation benchmark proposed in the WBS paper (Fryzlewicz 2012) page 29. In that paper 5 scenarios are considered. We considered an additional scenario from a further paper on SMUCE (Futschik et al. 2014) corresponding to Scenario 2 of WBS with a standard deviation of 0.2 rather than 0.3. We call this Scenario 2’. We first compared FPOP with $\beta =2 \log (n)$, WBS with the sSIC and SMUCE with $\alpha =0.45$ (used in Futschik et al. (2014) for Scenario 2’) in terms of mean squared error (MSE). For FPOP we first standardised the signal using the MAD (Mean Absolute Deviation) estimate as was done for PELT in Fryzlewicz (2012).

Using 2000 replications per scenario we tested the hypotheses

- $\mathbf{{H_0}}$ the average MSE difference between WBS and FPOP is lower or equal to 0.
- $\mathbf{{H_1}}$ the average MSE difference between WBS and FPOP is larger than 0.

using a paired t-test and paired Wilcoxon test. $H_0$ is clearly rejected (p value $< 10^{-16}$) in 4 scenarios out of the 6 (1, 2, 2$^\prime$ and 5). We did the same thing with SMUCE and we found that $H_0$ is rejected in 4 scenarios (1, 2, 4 and 5). The R code of this comparison is available on R-Forge.

More generally, we compared WBS with the sSIC, mBIC and BIC penalty, SMUCE with $\alpha =0.35$, 0.45 and 0.55 and FPOP with $\beta = \log (n)$, $2\log (n)$ and $3 \log (n)$. For each scenario we made 500 replications. We assessed the ability to recover the true number of changes $\hat{K}$, computed the mean squared error (MSE) and breakpoint error (BkpEr) from the breakpointError R package and counted the number of exactly recovered breakpoints (exact TP). With $\beta = 2\log (n)$ or $3\log (n)$ FPOP gets better results, in terms of MSE, $\hat{K}$, exact TP and BkpEr, than SMUCE and WBS in Scenarios 1 and 5. WBS is better than FPOP and SMUCE in Scenario 4. In Scenarios 2 and 3 WBS and FPOP are comparable (WBS is better in terms of BkpEr and worst in terms of MSE). In Scenario 2’ FPOP and SMUCE are comparable. The average of each approach is given in a supplementary data file, and the R code is available on R-Forge in the “benchmark wbs” directory.

We performed similar analysis on our speed benchmark (Fig. 7, left) and found that FPOP is competitive or better than WBS and SMUCE in terms of MSE, BkpEr, exact TP and $\hat{K}$. Results are shown in supplementary file. The R codes are also available on R-forge.

## Discussion

We have introduced two new algorithms for detecting changepoints, FPOP and SNIP. A natural question is which of these, and the existing algorithms, pDPA and PELT, should be used in which applications. There are two stages to answering this question. The first is whether to detect changepoints through solving the constrained or the penalised optimisation problem, and the second is whether to use functional or inequality based pruning.

The advantage of solving the constrained optimisation problem is that this gives exact segmentations for a range of numbers of changepoints. The disadvantage is that solving it is slower than solving the penalised optimisation problem, particularly if there are many changepoints. In interactive situations where you wish to explore segmentations of the data, then solving the constrained problem is to be preferred (Hocking et al. 2014). However in non-interactive scenarios when the penalty parameter is known in advance, it will be faster to solve the penalised problem to recover the single segmentation of interest. Further, recent work in Haynes et al. (2014) explores a way of outputting multiple segmentations (corresponding to various penalty values) for the penalised problem.

The decision as to which pruning method to use is purely one of computational efficiency. We have shown that functional pruning always prunes more than inequality based pruning, and empirically have seen that this difference can be large, particularly if there are few changepoints. However functional pruning can be applied less widely. Not only does it require a stronger condition on the cost functions, but currently its implementation has been restricted to detecting changes in a univariate parameter from a model in the exponential family. Even for situations where functional pruning can be applied, its computational overhead per non-pruned candidate is higher.

Our experience suggests that you should prefer functional pruning in the situations where it can be applied. For example FPOP was always faster than PELT for detecting a change in mean in the empirical studies we conducted, the difference in speed is particularly large in situations where there are few changepoints. Furthermore we observed FPOP’s computational speed was robust to changes in the number of changepoints to be detected, and was even competitive with, and sometimes faster than, Binary Segmentation.

*Software* C++ implementation (within an R wrapper) for the FPOP algorithm can be found in the opfp project repository on R-Forge: https://r-forge.r-project.org/R/?group_id=1851.

*Reproducibility* The subversion repository of the opfp project on R-Forge contains all the code necessary to make the figures in this manuscript.

### Electronic supplementary material

Below is the link to the electronic supplementary material.

Supplementary material 1 (pdf 19 KB)

## Short description of the WBS benchmark scenarios

The 6 scenarios considered in Sect. 7.4 are:

- (1) block profiles of length 2048. Changepoints are at 205, 267, 308, 472, 512, 820, 902, 1332, 1557, 1598, 1659. Means are 0, 14.64, $-$3.66, 7.32, $-$7.32, 10.98, $-$4.39, 3.29, 19.03, 7.68, 15.37, 0. The standard deviation is 10.
- (2) fms profiles of length 497. Changepoints are at 139, 226, 243, 300, 309, 333. Means are $-$0.18, 0.08, 1.07, $-$0.53, 0.16, $-$0.69, $-$0.16. The standard deviation is 0.3.
- (2$^\prime$) fms$^\prime$, profiles of length 497. Changepoints are at 139, 226, 243, 300, 309, 333. Means are $-$0.18, 0.08, 1.07, $-$0.53, 0.16, $-$0.69, $-$0.16. The standard deviation is 0.2.
- (3) mix profiles of length 560. Changepoints are at 11, 21, 41, 61, 91, 121, 161, 201, 251, 301, 361, 421, 491. Means are 7, $-$7, 6, $-$6, 5, $-$5, 4, $-$4, 3, $-$3, 2, $-$2, 1, $-$1. The standard deviation is 4.
- (4) teeth10 profiles of length 140. Changepoints are at 11, 21, 31, 41, 51, 61, 71, 81, 91, 101, 111, 121, 131. Means are 0, 1, 0, 1, 0, 1, 0, 1, 0, 1, 0, 1, 0, 1. The standard deviation is 0.4.
- (5) stairs 10, profiles of length 150. Changepoints are at11, 21, 31, 41, 51, 61, 71, 81, 91, 101, 111, 121, 131, 141. Means are 1, 2, 3, 4, 5, 6, 7, 8, 9, 10, 11, 12, 13, 14, 15. The standard deviation is 0.3.
