# Supplementary material for: On optimal multiple changepoint algorithms for large data
Source: Stat Comput. 2016 Feb 15;27(2):519–33. doi: 10.1007/s11222-016-9636-3 (PMC7175693; doi:10.1007/s11222-016-9636-3)
Supplement: Supplementary file 1 — Supplementary material 1 (pdf 19 KB) [file 11222_2016_9636_MOESM1_ESM.pdf]

Supplementary Table 1: Performance of WBS, FPOP and SMUCE in our speed simulations with 50 replications. Khat : Proportion of true K recovery, MSE : average mean square error, exactTP and FP (average number of exactly recovered and false breakpoint), BkpE : average breakpointError (see R package)

WBS_1

	Khat	MSE	exactTP	exactFP	BkpE
WBS.ssic	0.52	2.60E-2	4.40	7.15	1.12
WBS.bic	0.54	2.58E-2	4.41	7.16	1.11
WBS.mbic	0.41	2.73E-2	4.35	6.99	1.23
Fpop.3	0.20	2.89E-2	4.33	6.65	1.45
Fpop.2	0.57	2.44E-2	4.50	7.10	1.06
Fpop.1	0.05	4.45E-2	4.52	12.56	5.92
Smuce.0.55	0.02	3.65E-2	4.01	6.64	1.94
Smuce.0.45	0.01	4.03E-2	3.87	6.63	2.13
Smuce.0.35	0.01	4.26E-2	3.82	6.55	2.26

WBS_2

	Khat	MSE	exactTP	exactFP	BkpE
WBS.ssic	0.95	4.12E-2	4.10	2.93	0.27
WBS.bic	0.96	4.09E-2	4.11	2.94	0.27
WBS.mbic	0.95	4.29E-2	4.07	2.89	0.29
Fpop.3	0.87	4.71E-2	4.06	2.70	0.44
Fpop.2	0.94	3.81E-2	4.20	2.84	0.30
Fpop.1	0.14	9.21E-2	4.18	6.58	3.97
Smuce.0.55	0.76	5.71E-2	3.90	2.93	0.62
Smuce.0.45	0.70	6.30E-2	3.80	2.94	0.70
Smuce.0.35	0.64	6.81E-2	3.70	2.95	0.78

WBS_2'(sigma as in Smuce)

	Khat	MSE	exactTP	exactFP	BkpE
WBS.ssic	0.96	3.38E-2	4.90	2.15	0.12
WBS.bic	0.95	3.35E-2	4.91	2.14	0.12
WBS.mbic	0.97	3.29E-2	4.91	2.12	0.09
Fpop.3	0.99	2.97E-2	5.00	2.01	0.08
Fpop.2	0.95	3.07E-2	5.00	2.06	0.12
Fpop.1	0.15	8.21E-2	4.97	5.59	3.63
Smuce.0.55	0.95	3.00E-2	5.00	2.05	0.12
Smuce.0.45	0.97	2.98E-2	5.00	2.03	0.10
Smuce.0.35	0.98	2.97E-2	5.00	2.02	0.09

WBS_3

	Khat	MSE	exactTP	exactFP	BkpE
WBS.ssic	0.29	9.66E-2	7.30	5.59	1.94
WBS.bic	0.32	9.67E-2	7.27	5.70	1.89
WBS.mbic	0.15	1.04E-1	7.20	5.07	2.34
Fpop.3	0.06	1.19E-1	7.02	4.56	2.92
Fpop.2	0.32	9.65E-2	7.32	5.56	1.97
Fpop.1	0.09	1.32E-1	7.43	9.84	4.57
Smuce.0.55	0.08	1.15E-1	6.95	5.51	2.42
Smuce.0.45	0.05	1.25E-1	6.86	5.40	2.66
Smuce.0.35	0.02	1.36E-1	6.76	5.25	2.93

WBS_4

	Khat	MSE	exactTP	exactFP	BkpE
WBS.ssic	0.74	3.70E-1	9.21	4.44	1.84
WBS.bic	0.75	3.58E-1	9.33	4.46	1.75
WBS.mbic	0.43	8.69E-1	5.43	2.93	6.43
Fpop.3	0.18	9.24E-1	5.27	2.73	6.59
Fpop.2	0.62	4.24E-1	8.93	4.03	2.20
Fpop.1	0.38	3.56E-1	9.60	5.79	2.54
Smuce.0.55	0.02	1.19E+0	3.15	5.47	7.86
Smuce.0.45	0.01	1.23E+0	2.84	5.26	8.25
Smuce.0.35	0.01	1.29E+0	2.39	5.10	8.84

WBS_5

	Khat	MSE	exactTP	exactFP	BkpE
WBS.ssic	0.59	2.60E-1	12.19	3.35	1.02
WBS.bic	0.58	2.61E-1	12.19	3.37	1.05
WBS.mbic	0.62	2.62E-1	12.15	3.32	0.98
Fpop.3	0.94	2.29E-1	12.47	2.48	0.44
Fpop.2	0.95	2.15E-1	12.52	2.53	0.44
Fpop.1	0.39	2.63E-1	12.50	3.79	1.66
Smuce.0.55	0.15	1.17E+0	8.43	4.58	4.02
Smuce.0.45	0.10	1.33E+0	7.80	4.85	4.54
Smuce.0.35	0.07	1.45E+0	7.44	4.90	4.89

Supplementary Table 2 : Performance of WBS, FPOP and SMUCE in our speed simulations with 50 replications. Khat : Proportion of true K recovery, MSE : average mean square error, exactTP and FP : average number of exactly recovered breakpoint, BkpE : average breakpointError (see R package)

n= 200000, Bkp= 10

	Khat	MSE	exactTP	exactFP	BkpE
WBS.ssic	1.00	1.57E-4	5.70	4.30	0.00
WBS.bic	1.00	1.60E-4	5.66	4.34	0.00
WBS.mbic	1.00	1.61E-4	5.64	4.36	0.00
Fpop.3	1.00	1.56E-4	5.64	4.36	0.00
Fpop.2	1.00	1.56E-4	5.64	4.36	0.00
Fpop.1	0.00	6.55E-4	5.66	11.88	7.54
Smuce.0.55	0.86	1.58E-4	5.64	4.50	0.14
Smuce.0.45	0.94	1.56E-4	5.64	4.42	0.06
Smuce.0.35	0.96	1.56E-4	5.64	4.40	0.04

n= 200000, Bkp= 50

	Khat	MSE	exactTP	exactFP	BkpE
WBS.ssic	0.54	1.06E-3	30.36	19.60	1.45
WBS.bic	0.56	1.09E-3	30.28	19.34	1.12
WBS.mbic	0.46	1.12E-3	30.08	19.48	1.29
Fpop.3	0.72	8.79E-4	31.58	17.86	0.62
Fpop.2	0.84	8.45E-4	31.76	17.92	0.39
Fpop.1	0.00	1.43E-3	31.80	26.80	8.97
Smuce.0.55	0.68	8.80E-4	31.58	18.06	0.65
Smuce.0.45	0.70	8.80E-4	31.58	18.04	0.62
Smuce.0.35	0.72	8.80E-4	31.58	18.02	0.59

n= 200000, Bkp= 100

	Khat	MSE	exactTP	exactFP	BkpE
WBS.ssic	0.14	2.89E-3	57.44	41.08	5.18
WBS.bic	0.16	2.92E-3	57.22	41.78	5.82
WBS.mbic	0.14	2.92E-3	57.88	40.70	5.37
Fpop.3	0.38	1.87E-3	61.76	36.44	2.06
Fpop.2	0.58	1.78E-3	62.22	36.70	1.38
Fpop.1	0.04	2.32E-3	62.44	45.50	9.58
Smuce.0.55	0.36	1.89E-3	61.70	36.92	2.01
Smuce.0.45	0.36	1.91E-3	61.60	36.96	2.13
Smuce.0.35	0.36	1.91E-3	61.60	36.96	2.11

n= 200000, Bkp= 500

	Khat	MSE	exactTP	exactFP	BkpE
WBS.ssic	0.00	1.73E-2	264.94	230.38	84.81
WBS.bic	0.04	1.73E-2	265.20	229.88	83.55
WBS.mbic	0.02	1.78E-2	261.92	228.96	82.51
Fpop.3	0.00	1.18E-2	292.64	166.12	45.15
Fpop.2	0.00	1.00E-2	301.14	171.12	32.35
Fpop.1	0.08	9.85E-3	310.52	190.86	32.02
Smuce.0.55	0.00	1.38E-2	286.34	176.30	48.17
Smuce.0.45	0.00	1.42E-2	284.78	176.42	49.90
Smuce.0.35	0.00	1.47E-2	283.12	176.26	51.83

n= 200000, Bkp= 1000

	Khat	MSE	exactTP	exactFP	BkpE
WBS.ssic	0.00	3.56E-2	498.14	448.14	211.32
WBS.bic	0.00	3.55E-2	499.12	450.16	213.70
WBS.mbic	0.00	3.67E-2	495.04	441.60	207.83
Fpop.3	0.00	2.97E-2	537.54	300.38	172.82
Fpop.2	0.00	2.22E-2	572.26	320.98	120.18
Fpop.1	0.00	1.94E-2	605.82	363.10	86.42
Smuce.0.55	0.00	3.98E-2	502.74	338.88	196.30
Smuce.0.45	0.00	4.16E-2	497.04	338.76	202.65
Smuce.0.35	0.00	4.43E-2	490.00	337.82	211.98
